# Morbid Anatomy

**Published:** 1833-04-01

**Authors:** 


### WORKS ON MORBID ANATOMY.

1. ILLUSTRATIONS OF THE ELEMENTARY FORMS OF DISEASE. By *Robert Carswell*, M.D. Professor of Pathological Anatomy in the University of London, &c. &c. Fasciculus first—Tubercle. Royal 4to, four coloured Lithographic Plates, with accompanying Letter-press. London, Longman's, 1833.

2. PRINCIPLES AND ILLUSTRATIONS OF MORBID ANATOMY, &c. BEING A COMPLETE SERIES OF COLOURED LITHOGRAPHIC DRAWINGS, &c. &c. By *J. Hope*, M.D. F.R.S. Physician to the St. Mary-le-bone Infirmary, &c. Large 8vo. Part II. February, 1833, price 8*s*. 6*d*.

IN our last Number, we had occasion to speak in a very favourable manner of the first part of Dr. Hope's Illustrations of Morbid Anatomy. Since the publication of that part, Dr. Carswell has brought the first fasciculus of his work into the field, and both are now before the public. We will not attempt the invidious task of comparing them. They are both of considerable merit, and we hope there is scope for each.

The able and industrious authors have adopted different plans in their development of the subject. Dr. Hope takes up an organ or an apparatus, and displays all its lesions; Dr. Carswell, on the other hand, selects an elementary lesion or morbid formation, and traces it in various organs. Thus, Dr. Hope's first and second parts are devoted to the morbid changes observed in the pulmonary organs, whilst Dr. Carswell has selected tubercle as the subject of his first fasciculus, and shewn it in the lung, spleen, kidney, testis, uterus, intestine, &c. It will be seen that an important difference exists between the methods adopted by these gentlemen. Leaving any farther comparison, we will consider separately the fasciculi before us.

And first of that of Dr. Carswell. It is dedicated, as we have said, to the delineation and description of tubercle. To the delineations we shall allude bye and bye—at present, we will endeavour to give a brief account of his views of the origin and progress of tubercle.

Dr. Carswell gives the following definition of tuberculous matter, constituting, as it does, the essential anatomical character of those diseases now termed tubercular. It is a pale yellow, or yellowish-grey, opaque, unorganized substance, the form, consistence, and composition of which vary with the nature of the part in which it is formed, and the period at which it is examined. Dr. C. next considers, seriatim, the seat, form, consistence, and colour, composition, softening, and progress of tuberculous matter.

*Seat of Tuberculous Matter.* Of all organs, and of all tissues, the mucous membrane is most especially the seat of this morbid production. Thus, it is found in the mucous system of the respiratory, digestive, biliary, urinary, and generative organs. It is also observed on the secreting surface of serous membranes, particularly the pleura and peritoneum, and in the cells of the cellular tissue; it often forms considerable accumulations in the lacteals and lymphatics, both before and after they unite to form their respective glands; it is also seen in the substance of the brain and cerebellum, in accidental cellular tissue, and in the blood.

*Form of Tuberculous Matter.* The round form which it ordinarily assumes is, Dr. Carswell thinks, accidental, and arises from the equal opposition offered on all sides to its development. In parts where such equal opposition is not offered, that round form is not maintained. Thus, if confined to the secreting surface, it assumes the form of a shut or open globular sac—in the air-cells it is globular—in the bronchi, of a tubular or cylindrical form, with a ramiform distribution, terminated by a cauliflower arrangement of the air-cells; in short, it assumes, in various tissues or organs, the form of the containing parts. When the secretion of tuberculous matter takes place, so as to become disseminated throughout a considerable extent of an organ, it is said to be infiltrated, and has no definite form, save that of the part in which it is diffused. The granular arrangement of tuberculous matter in the lungs, is owing to its accumulation in one or more contiguous air-cells, and the lobular character which it occasionally presents in the same organ, is due to its being confined in the air-cells of a single lobule.

*Consistence and Colour.* "Tuberculous matter does not acquire its maximum of consistence until an indefinite period after its formation. It is frequently found in its primitive state in the bronchi, air-cells, biliary ducts and their dilated extremities, in the cavity of the uterus and fallopian tubes, &c. resembling a mixture of soft cheese and water, both in consistence and colour; but when much resistance is offered to its accumulation, as in the lymphatic glands, and even sometimes in the air-cells of a whole lobule, it may feel as firm as liver or pancreas. These extreme degrees of consistence of tuberculous matter depend not only on the resistance which the tissues of these and other parts oppose to its accumulation, but also on the removal of its watery part some time after it has been deposited. Hence it follows that tuberculous matter may, when first perceived, be either very soft or remarkably firm. In the first case it is pultaceous, and feels somewhat granular when rubbed between the fingers; in the second, friable: and in both it is of a pale yellow colour, and opaque.

The grey semi-transparent substance, already alluded to, by no means necessarily precedes the formation of the pale yellow or opaque tuberculous matter; it is, indeed, observed in a few only of the many organs in which the latter is found. Thus it is never seen in the cavity of the uterus or fallopian tubes; in the ureters, pelvis, or infundibula of the kidneys; in the mucous follicles of the intestines; in the lacteals or lymphatics; in the biliary ducts; nor do I recollect to have seen it in the cerebral substance. I have never met with it in the bronchi, unless in some of their most minute or terminal branches. On the contrary, the semi-transparent substance is frequently seen in the air-cells and on the free surface of serous membranes, particularly the peritoneum; and in both it is certainly sometimes observed to *precede* the formation of opaque tuberculous matter; because, first, a number of cells of the same lobule are seen filled with the former, whilst the remaining cells contain the latter substance; secondly, because on the peritoneum the grey semi-transparent substance is generally more abundant than the pale yellow opaque matter; and, thirdly, because a small nucleus of the latter is frequently enclosed in a considerable quantity of the former. The following is the explanation which I would offer of these exceptional conditions to the regular and ordinary formation of the tuberculous matter. But, first of all, it is necessary to remark that the formation and manifestation of this matter as a morbid product cannot take place unless the fluid from which it is separated—the blood—has been previously modified. This important fact being admitted for the present, it is obvious that a healthy secreting surface may separate from the blood not only the materials of its own peculiar secretion, but also those of tuberculous matter. Such is, indeed, what takes place in the air-cells. The mucous secretion of their lining membrane accumulates where it is formed; but it is not pure mucus; it contains a quantity of tuberculous matter mixed up with it, which after a certain time is separated, and generally appears in the form of a dull, yellow opaque point, occupying the centre of the grey, semi-transparent, and, sometimes, inspissated mucus. This process of separation of tuberculous matter from secreted fluids is strikingly exemplified in tubercular peritonitis. When we examine the peritoneum thus affected, the three following stages of the process are frequently extremely well marked: first, on one portion of this membrane there is seen a quantity of recently secreted coagulable lymph; secondly, on another we find the same plastic semi-transparent substance, partly organized, and including within it, or surrounding a globular mass of tuberculous matter; and, lastly, on another part the coagulable lymph is found converted into a vascular or pale cellular tissue, covered by an accidental serous membrane, beneath which, and external to the peritoneal or original secreting surface, the tuberculous matter is seated, having the form of a round granular eminence, resembling in colour and consistence pale firm cheese.

In this as well as in the preceding case we cannot but perceive that the formation of tuberculous matter originates in a process similar to that of secretion; that its separation from the blood may be accompanied with that of natural and also other morbid secretions; and hence the reason why its physical characters are sometimes obscured, particularly in the first stage of its formation."

*Composition of Tuberculous Matter.* Its chemical composition varies at different periods, in different animals, and probably in different organs. In man it is chiefly composed of albumen, with various proportions of gelatine and fibrine; in the cow it contains a large proportion of earthy salts, in which the phosphate of lime is said either to predominate, or to exist in the same proportion, along with the carbonate of lime, as it does in bones. Dr. Carswell insists on the proposition, that tuberculous matter is capable of undergoing no change, save what is induced in it by the influence of external agents.

*Softening of Tuberculous Matter.* Our readers are aware that the mode of softening has been a litigated question with morbid anatomists. Laennec said that the softening commenced in the centre. Andral denied this, and maintained that no softening occurs but what may be attributed to the mixture of pus or other fluids. This is supported by Dr. Carswell; he endeavours to explain the apparent central softening which is thought to have misled Laennec. When tuberculous matter is found in the lungs, it is generally contained in the air-cells and bronchi. If the morbid product be confined to the surface, or has accumulated so as to leave only a limited central portion of their cavities unoccupied, it is obvious that, on a transverse division, a bronchial tube will resemble a tubercle, having a central depression or soft central point, because, in the centre, there is no tuberculous matter, but merely a small quantity of mucus, or other secreted fluids; the air-cells will also exhibit a number of similar appearances, or rings of tuberculous matter, grouped together, and containing in their centre a quantity of similar fluids. Softening usually takes place at the circumference of firm tuberculous matter, in consequence of its proving an irritant to the inclosing tissues, which ulcerate and secrete pus, &c. This is the reason why softening is frequently seen making its appearance in several points of an agglomerated mass of this substance, which has included within it portions of the tissues in which it was formed.

Dr. Carswell doubts whether tubercle be ever really *encysted;* or, at least, he believes that this term is almost always incorrect. The walls of the air-cells in the lungs, the extremities of the biliary system in the liver, &c. are mistaken for cysts.

*Progress and Termination of Tuberculous Matter.* Dr. Carswell limits himself here to the manner in which tuberculous disease is cured. Scrofulous enlargement of glands does, as Dr. C. believes, disappear—tabes mesenterica has been known to terminate favourably—and Dr. Carswell has traced the curative process in the bronchial glands and in the lungs. The natural process of cure, or progress towards it, would seem, according to Dr. C. to consist of the conversion of the tuberculous matter into a cretaceous substance, or one resembling a mixture of putty and dried mortar. The bronchial glands frequently excite inflammation, adhesion, and ulceration in a contiguous part of the trachea, and the morbid matter is discharged into this tube. We will transcribe Dr. Carswell's history of the cure of tubercular phthisis.

"The tuberculous matter whether contained in a bronchial tube, the air-cells, or cellular tissue of the lungs, has assumed a dry, putty-looking, chalky, or cretaceous character. If these changes are observed in an excavation, the surrounding pulmonary substance is generally dark-coloured; and if the excavation exists in the course of large bronchial tubes, those situated between the excavation and the periphery of the lungs are obliterated, whilst those in the opposite direction terminate either in a shut extremity near the excavation, or are continuous with the lining membrane or accidental tissue which encloses the altered tuberculous matter. The existence of this accidental tissue is an important circumstance as regards the cure of tubercular excavations. It is formed by the effusion of coagulable lymph on the surface of the excavation, or in the substance of the contiguous pulmonary tissue; has at first, and so long as a ready exit is afforded to its secretion, the characters of simple mucous tissue; but at a later period, and especially when the latter condition is wanting, it becomes gradually and successively converted into serous, fibrous, fibro-cartilaginous, and cartilaginous tissues.

The cartilaginous and the osseous transformations of this accidental tissue are, however, rare, particularly the latter. It much more frequently retains the fibrous character, and possesses the property of contracting so as to diminish the bulk of the excavation, and carry with it the pulmonary tissue with which it is connected. The diminution of bulk which accompanies the removal of the tuberculous matter, and the contraction of this accidental tissue, give rise to a puckering of the lung, which is best seen where the pleura is forced to follow the retrocession of the pulmonary substance beneath it, and around what is called the cicatrix: for there sometimes remains only a small globular, oval, or even linear portion of fibrous or fibro-cartilaginous tissue, in a part of the lung, where, from the extensive puckering around it, there must have formerly existed an excavation of considerable extent."

The foregoing is a tolerably complete account of the history of tubercle, as given by Dr. Carswell. We have frequently remarked, with some surprise, that morbid anatomists, they who advocate exactness, and the nature of whose science would seem to admit of it, are extremely theoretical. Look at the views of Dr. Baron, of Dr. Hodgkin, and of other able and distinguished writers. The truth is, that, not content with cognizable facts, they are anxious, and naturally anxious, to go further than Nature, we fear, will permit. The search for the primary development of morbid products—for the manner in which they are deposited—the exact tissue in which they are seated, is, like the hunt for the ultimate fibre of muscle, or the ultimate globule of the brain, a painful and a fruitless one.

We make these observations not to derogate in any way from the merits, the ingenuity, or the accuracy of Dr. Carswell. We do not pretend to pronounce an opinion on the truth or otherwise of some of his notions respecting tubercle; yet it would be a want of candour on our part, not to say that we have some misgivings with respect to their absolute correctness. We should not be surprised if some morbid anatomist were to criticise his conclusions as to the seat of tubercle, and deny that it is where he places it. But, waiving this, we think Dr. Carswell's history of this morbid production well worthy the attention of our readers.

The fasciculus before us contains four coloured lithographic plates, each containing some four or five drawings. In the first plate is represented tuberculous matter, deposited in the air-cells of the lung and in the bronchi —in the second, it is seen in the uterus, the kidney, the rabbit's liver, the testis—in the third, it is displayed in the lacteals and mesenteric glands, the cerebrum and cerebellum, the spleen, and in accidental cellular tissue covering the intestine—in the fourth, tuberculous infiltration of the lung is delineated, with the progress of a vomica towards cure, &c. Of the execution of the drawings we can speak highly, and we do not doubt that they are as accurate as they are beautiful. We do not conceive that any lengthened encomium is necessary, to make our readers anxious to be enabled to judge for themselves. We trust that Dr. Carswell will meet with all the success he deserves, and we feel assured that that is not trifling.

We must next advert to the second fasciculus of Dr. Hope's work, and we fear that our lengthened view of Dr. Carswell's must cripple us in our notice of this.

It will be recollected that Dr. Hope took up, in his first fasciculus, the consideration of the diseases of the pulmonary apparatus. In the present fasciculus he continues that subject, and, as in that he treated of peripneumony and pleurisy, in this he passes to phthisis pulmonalis, pulmonary apoplexy, emphysema, encephaloid tumour, melanosis, some minor alterations, and bronchitis. There is more letter-press in this than in the former part, and the delineations are of a superior rather than an inferior description. There are four plates and twenty-three figures. The latter are, on the whole, more highly coloured, perhaps more highly finished, than their predecessors, and, from not being of so determinate a figure, have a less formal air. We will not attempt to single out particular figures for particular encomium, nor particular criticism; we recommend the public to judge for themselves of these publications. The matter contained in the letter-press is fitter for our notice, and we shall endeavour to single out such portions as may seem instructive.

Dr. Hope commences with a notice of tubercle, that production which has occupied, as we have seen, the attention of Dr. Carswell. Dr. H. notices the theories of its primary state; he points out the objections that have been made to all, and he offers on his own part none. Perhaps this is prudent; perhaps it is philosophical to commence exact description where observation itself begins to be exact, and not to pave a system of facts with conjectures.

Tubercles may enlarge; but how? from within or from without? Reasoning would say from without, and facts appear to prove it—appear to shew, that the increase is by juxta-position of fresh particles of tuberculous matter, secreted by the tunics around. Drawings displaying this are given by Dr. Hope.

"The mode of tubercular deposition, therefore, may, in conformity with the above illustrations, be explained in the following way. In the spot where the tubercular secretion has commenced, each *living* molecule, instead of its natural exhalation, separates from the blood a molecule of tubercular matter, which, superadded to the molecules already secreted, contributes to increase their mass. Thus, then, every tubercle is infiltrated into the tissues where it exists. Sometimes vestiges of these tissues may still be recognized in the substance of the tubercular mass, as already shewn in Fig. 17, *b*, Fig. 21, *e*, and Fig. 23; and to them are referrible the vessels which occasionally exist in the interior of tubercles. Sometimes the tissues totally disappear by compression. (Précis, i. 414.)" 28.

Tubercle, it is well known, may be either in conglomerated masses, when it is said to be infiltrated, or in isolated depositions. The former, Dr. Hope very justly observes, argues a highly tubercular diathesis, and is much more rapidly fatal than the latter.

The final transformations of tubercle are, 1, cretaceous induration; 2, softening by suppuration. We need not allude to the description of the former; its appearance must be familiar. Dr. H. has occasionally seen cretaceous tubercles in a softened state. In general they have been of a large size, from that of a marble to that of a walnut, existing in a very limited number, and often in otherwise healthy lung. They consisted of softish flakes and grains of friable calcareous matter, intermixed with a very little purulent fluid.

Softening is believed by Dr. Hope to be occasioned by the secreted pus, acting on the tuberculous matter exposed to its influence. In his remarks on the liability of vomicæ, situated near the pleura, to ulcerate through it, Dr. Hope remarks that nature guards against this in three ways: by exciting chronic inflammation and fibro-cartilaginous thickening of the sub-serous cellular tissue, or an albuminous deposition like white of egg, two-thirds boiled, which eventually hardens into fibro-cartilaginous tissue—by causing the formation of a dense fibrous false membrane on the serous surface of the pleura—or, finally, by causing adhesions of the pulmonary to the costal pleura.

The first drawing in this fasciculus is one of a tuberculous cavern that appears to have been cured. We saw the case, and can vouch for the accuracy of the memorandum and of the delineation. The cavity exceeded the size of the largest orange, and occupied nearly the whole of the upper lobe of the right lung.

*Case.* "A female, æt. 40, at St. George's, under Dr. Hewitt, presenting a remarkable instance of temporary recovery from consumption. Four years before admission she had hæmoptysis after a fall, followed by hectic, emaciation, and all the symptoms of confirmed phthisis. These subsided, and she was able to resume her accustomed avocations. They subsequently recurred, and, on admission, she was emaciated, had cough, thick yellow opaque sputa, perspirations, and loud pectoriloquy over the whole upper lobe of the left lung. She had also the symptoms of organic disease of the heart, and died dropsical.

On dissection, the above described cavity was found, and the lung beneath it was dense, contained numerous small tubercles, and was deeply coloured with much black and grey pulmonary matter. A few tubercles in the apex of the opposite lung. The tubercular disease had probably recurred subsequent to the healing of the cavern." 65.

Dr. Hope observes that tubercle is most commonly formed in the cellular tissue. The organs most frequently affected in the adult, are, first, the lungs, next the ileum, next the mesenteric glands, next the great intestine, &c. It is rare, in adults, for tubercles to exist in other organs while there are none in the lungs. In infants and children, the order of frequency is different; it is, first, the bronchial glands, then the lungs, then the mesenteric glands, then the spleen, then the intestines, &c.

"With respect to frequency at different ages, observation shews, that prior to the age of fifteen, tubercles are, beyond comparison, the most common between the fourth and fifth year, three-fourths of those who die presenting, according to the tables of Lombard, more or less tubercular disease in various organs. Between the fifth and fifteenth year the disease is more frequent than before the fourth. After the fifteenth it increases; only, however, in the lungs, intestines, and mesenteric glands. Hence, after this age, it has acquired the name of pulmonary consumption.

From eighteen to forty, it is less common, though less so than from four to five. Females are more subject to it before the age of twenty, and males between twenty-one and twenty-eight. In the lower classes of working people, who are ill-fed and much exposed, I have found, from extensive observation in the Edinburgh and St. Marylebone Infirmaries, that tubercle is very common even up to the fiftieth year." 37.

Respecting pulmonary apoplexy, Dr. Hope observes that organic disease of the heart is by far its most frequent cause, two-thirds of the cases he has examined having been referrible to it. Contraction of the mitral valve, with hypertrophy of the right ventricle, is the lesion which he has found most uniform. This has been the case with the majority of patients, affected with pulmonary apoplexy, that we have seen examined after death.

We pass to Dr. Hope's observations on bronchitis, and we are tempted to extract his remarks on the different causes of *redness* of the mucous membrane.

"Chronic redness of the mucous membrane is more of a purplish, livid, violet, or deep brownish cast, all of which tints may be seen in the dilated bronchi, Figs. 50 and 52. In chronic bronchitis, especially if purulent, the mucous membrane is, in a few instances, perfectly pale.

In the larynx, trachea, and great bronchi, redness generally proceeds from inflammation; in the minute bronchi, it is frequently an effect of mechanical engorgement during life, or of sanguineous gravitation after death. Redness from all these causes must be distinguished from that occasioned by putrefaction; nor must it be confounded with the natural redness of the lungs apparent through the walls of the finer bronchial tubes.

Redness of the mucous membrane is not *necessarily* excited by the diseases of the pulmonary substance. Though generally present in peripneumony, it is occasionally absent. Unsoftened tubercles sometimes exist, even in abundance, without giving rise to it, but its absence is very rare when they have formed excavations. Hence the principal part of the irritation and expectoration in phthisis results from bronchitis. The red is generally deeper in the vicinity of the caverns." 57.

Dr. Hope observes that thickening of the mucous membrane is a consequence of bronchitis, and that this thickening may result from two conditions; 1, sanguineous engorgement—2, hypertrophy. We merely notice this to afford ourselves an opportunity of alluding to a case from which a drawing has been made. The case itself has been taken from Andral, and is an instance of contraction from thickening of the mucous membrane.

"A toyman, æt. 31, had the symptoms of organic disease of the heart. Said that he had long experienced a sort of constriction a little above the right nipple; and that he did not breathe on the right side of the chest. The respiration behind was very strong, with a mixture of mucous ronchus: in front, on the left, it was similarly loud, but on the right, under the clavicle, the inspiratory murmur, though pure, was much more feeble than on the left, yet there was no dulness on percussion. Hence emphysema was expected. Hydrothorax, from the disease of the heart, supervened, and was fatal.

*Sectio.*—The superior lobe of the right lung presented no emphysema, but its tissue was very deficient in crepitancy, though otherwise sound. The principal bronchus presented, a few lines from its origin, a contraction so great that a fine stiletto could scarcely pass through it. A little before dividing, the bronchus regained its usual calibre. Over the extent of the contraction, the fibrous coat was natural, but the mucous membrane was red and greatly thickened. The bronchi and lungs were otherwise healthy. Hydrothorax; hypertrophy with dilatation; contracted aorta." xvi.

The only other topic to which we shall allude is ulceration of the pulmonary mucous membrane. It may affect the larynx, trachea, or bronchi, but is most common in the first, and is rarely seen there unconnected with phthisis; that is, idiopathic laryngeal phthisis is extremely rare. Any part of the larynx may be affected, and the ulcers may present every variety with respect to number, size, and form. They are less common in the trachea, and still less so in the bronchi. They may result from acute as from chronic inflammation; they are not rare in variola. Ulcers are commonly limited in depth by occasioning deposition into the sub-mucous cellular tissue, but sometimes they cause perforation into the contiguous parts. Perforation, however, more frequently commences externally, in consequence of pulmonary vomicæ, tubercular bronchial glands, diseases of the aorta, œsophagus, and thyroid gland.

Here we must conclude, and we can only repeat, that we wish well to both the gentlemen whose works we have been noticing. Both are able, both industrious, both we have the honour of knowing, and the pleasure of esteeming. It is for the public to decide on their comparative merits, and we trust that the public will afford encouragement to either. As future fasciculi appear we shall introduce them to our readers.

